# Liraglutide treatment improves the coronary microcirculation in insulin resistant Zucker obese rats on a high salt diet

**DOI:** 10.1186/s12933-020-01000-z

**Published:** 2020-02-24

**Authors:** Vijayakumar Sukumaran, Hirotsugu Tsuchimochi, Takashi Sonobe, Mark T. Waddingham, Mikiyasu Shirai, James T. Pearson

**Affiliations:** 1 Department of Basic Medical Sciences, College of Medicine, Member of QU Health, Qatar University, Doha, Qatar; 2 Department of Cardiac Physiology, National Cerebral and Cardiovascular Center Research Institute, Suita, Osaka 564-8565 Japan; 3 Department of Physiology, Monash Biomedicine Discovery Institute, Monash University, Clayton, 3800 Australia; 4 Department of Pharmacology, College of Medicine, Member of QU Health, Qatar University, Doha, Qatar; 5 Department of Advanced Medical Research in Pulmonary Hypertension, National Cerebral and Cardiovascular Center Research Institute, Suita, Osaka 564-8565 Japan

**Keywords:** Endothelial dysfunction, Inflammation, Liraglutide, Nitric oxide, Oxidative stress

## Abstract

**Background:**

Obesity, hypertension and prediabetes contribute greatly to coronary artery disease, heart failure and vascular events, and are the leading cause of mortality and morbidity in developed societies. Salt sensitivity exacerbates endothelial dysfunction. Herein, we investigated the effect of chronic glucagon like peptide-1 (GLP-1) receptor activation on the coronary microcirculation and cardiac remodeling in Zucker rats on a high-salt diet (6% NaCl).

**Methods:**

Eight-week old Zucker lean (+/+) and obese (fa/fa) rats were treated with vehicle or liraglutide (LIRA) (0.1 mg/kg/day, s.c.) for 8 weeks. Systolic blood pressure (SBP) was measured using tail-cuff method in conscious rats. Myocardial function was assessed by echocardiography. Synchrotron contrast microangiography was then used to investigate coronary arterial vessel function (vessels 50–350 µm internal diameter) in vivo in anesthetized rats. Myocardial gene and protein expression levels of vasoactive factors, inflammatory, oxidative stress and remodeling markers were determined by real-time PCR and Western blotting.

**Results:**

We found that in comparison to the vehicle-treated fa/fa rats, rats treated with LIRA showed significant improvement in acetylcholine-mediated vasodilation in the small arteries and arterioles (< 150 µm diameter). Neither soluble guanylyl cyclase or endothelial NO synthase (eNOS) mRNA levels or total eNOS protein expression in the myocardium were significantly altered by LIRA. However, LIRA downregulated Nox-1 mRNA (*p *= 0.030) and reduced ET-1 protein (*p *= 0.044) expression. LIRA significantly attenuated the expressions of proinflammatory and profibrotic associated biomarkers (NF-κB, CD68, IL-1β, TGF-β1, osteopontin) and nitrotyrosine in comparison to fa/fa-Veh rats, but did not attenuate perivascular fibrosis appreciably.

**Conclusions:**

In a rat model of metabolic syndrome, chronic LIRA treatment improved the capacity for NO-mediated dilation throughout the coronary macro and microcirculations and partially normalized myocardial remodeling independent of changes in body mass or blood glucose.

## Background

Obesity, hypertension and prediabetes contribute greatly to coronary artery disease, heart failure and vascular events, and thus the morbidity and mortality attributable to cardiovascular disease in modern societies. Overweight and obese adults in comparison to their non-obese counterparts, have a greater susceptibility for subsequently developing diabetes mellitus and hypertension. Obesity leads to insulin resistance, vascular oxidative stress, reduced availability of vascular nitric oxide (NO), endothelial and vasomotor dysfunction of the coronary microcirculation, which contributes greatly to the development of cardiovascular complications [1–3]. Numerous lines of evidence over recent decades from epidemiological and experimental studies have clearly established that high Na^+^ intake not only contributes to elevated blood pressure, but also adverse cardiovascular remodeling, leading to the frequently reported higher incidence of cardiovascular events [4]. In common with obesity and diabetes, chronic high Na^+^ intake has been shown in part to drive vascular and myocardial remodeling independent of hypertension. Chronic exposure to a high Na^+^ diet was shown to impair aortic and mesenteric endothelial function in healthy Sprague–Dawley rats independent of blood pressure alterations [5, 6]. Similar findings have been observed in mice [7]. A combination of factors including vascular inflammation, an imbalance in redox state leading to excess oxidative stress, activation of the renin–angiotensin–aldosterone and sympathetic nervous systems are involved in the cardiovascular remodeling associated with chronic exposure to high Na^+^ intake, obesity and diabetes, and this is often shown to be compounded by the combination of these factors. Importantly, increased endothelial cell stiffness, inflammation and oxidative stress, and the impairment of endothelial function were shown to be mediated through upregulation of transforming growth factor beta (TGF-β1) following chronic high Na^+^ intake [8]. Prediabetic rats showed an exaggerated increase in peripheral perivascular inflammation and endothelial dysfunction to a high Na^+^ diet (6% NaCl) compared to normal Na^+^ diet and was associated with cardiac hypertrophy [9]. In that same study, angiotensin type 1 receptor blockade with valsartan was only partially effective in preventing the development of vascular damage but did not reduce cardiac hypertrophy induced by the high Na^+^ diet.

Glucagon-like peptide-1 (GLP-1) agonists have been recently approved as new therapeutic agents for patients with diabetes, based on evidence that GLP-1 agonists can stimulate glucose dependent insulin secretion from the β-cells of pancreatic islets [10]. The LEADER clinical study [Liraglutide Effect and Action in Diabetes: Evaluation of cardiovascular outcome Results] revealed that liraglutide (LIRA) treatment reduces mortality rates of cardiovascular events in diabetic patients [11]. LIRA treatment shown greater reductions in HbA1c in patients with cardiovascular events compared with placebo [12]. In addition, LIRA administration was more effective than metformin in suppressing vascular cell adhesion molecule (VCAM)-1 levels and reducing non-esterified free fatty acids recent-onset T2DM [13]. Indeed, previous studies have reported that GLP-1 agonist treatment has anti-inflammatory effects on peripheral vascular endothelial cells by increasing NO production and inhibiting cellular oxidative stress and apoptosis in diabetic animal models [14–16]. The chronic effects of GLP-1 agonists on the coronary circulation are less clear. The GLP-1 agonist exenatide reduced coronary microvessel barrier dysfunction in vivo in streptozotocin-treated rats and oxidative stress and apoptosis in endothelial cells cultured under high glucose conditions [17]. Systemic infusion of GLP-1 increased cardiac microvascular blood volume and velocity in healthy human subjects [18]. Nonetheless, short term treatment with LIRA had no significant effect on coronary flow reserve or peripheral microvessel endothelial function in type 2 diabetes mellitus (T2DM) patients [19]. However, it was recently suggested that improved left ventricular (LV) function and reduced in arterial stiffness following 6 months of LIRA treatment is attributable to a reduction in oxidative stress in newly diagnosed T2DM patients [20]. Whether chronic LIRA treatment can prevent the progression of endothelial dysfunction in the coronary circulation and adverse myocardial remodeling associated with metabolic syndrome has not been established. Recently, we showed that chronic LIRA treatment ameliorated the exacerbation of renal dysfunction and renal endothelial dysfunction in the Zucker obese rat model of metabolic syndrome compounded by high Na^+^ diet intake [21]. Since high-salt intake has been shown to potentiate cardiovascular inflammation and oxidative stress we hypothesized that chronic LIRA treatment of young adult Zucker obese rats on high-salt diet also prevents the development of inflammation, oxidative stress, coronary endothelial dysfunction and the pathological remodeling of the myocardium.

## Materials and methods

### Animal and experimental protocol

The study was approved by the National Cerebral and Cardiovascular Centre animal research committee (Approval number: 16079) and adhered for the “Guide for the Care and Use of Laboratory Animals” published by the US National Institutes of Health. Twenty-four male rats, Zucker lean (+/+, n = 12) and Zucker obese (fa/fa, n = 12) rats were purchased from SLC, Japan. Zucker +/+ and fa/fa rats were divided into two groups each to receive either saline or LIRA [0.1 mg/kg/day; 2ML-4, Alzet, CA, USA] for 8 weeks starting at the age of 8 weeks. Rats were provided with ad libitum access to normal drinking water and fed with high-Na^+^ chow diet (6% NaCl w/v, Oriental Yeast Co., Ltd, Tokyo, Japan). Liraglutide (VICTOZA) was purchased from Japan (Suzuken Co., Ltd., Tokyo, Japan).

### Glomerular filtration rate (GFR) measurements in conscious rats

The non-invasive clearance (NIC) kidney device (Mannhein Pharma & Diagnostics, Mannheim, Germany) was used to measure the GFR transcutaneously in Zucker rats. The device was affixed on a depilated region of the back using a double-sided patch during brief anesthesia with 2% isoflurane soon after induction. Fluorescein isothiocyanate (FITC)-sinistrin (7.5 mg/100 g bw, dissolved in saline; Fresenious Kabi, Linz, Austria) was administered via tail-vein injection and the rats were then placed in a metabolic cage for 2 h before downloading the recorded signals from the NIC-kidney device [22].

### Determination of renal function

Urinary levels of albumin, creatinine and albumin-creatinine ratio were measured in a single rapid colorimetric assay format using a Spectrum Diagnostic kit (Ref: 06072885-6010A, Siemens Vantage 2000 analyzer, Erlangen, Germany) according to the manufacturer’s protocol. Serum albumin and blood urea nitrogen (BUN) was measured by colorimetric qualitative analysis using commercially available Fuji dry-chem slides (DRI-CHEM 7000i, Fujifilm, Tokyo, Japan).

### Blood pressure measurement and echocardiography

Systolic blood pressure (SBP) was measured every 2-weeks in conscious animals by tail-cuff plethysmography method (98A-L, Softron Corporation, Tokyo, Japan) in 36–37 °C pre-heated chambers for about 10–15 min following training sessions prior to recording. After 8 weeks of high-salt diet, rats were subjected to transthoracic echocardiography during isoflurane anesthesia (2%) as previously described [23]. Briefly, intraventricular septal thickness at end-diastole (IVSd), intraventricular septal thickness at end-systole (IVDs), left ventricular (LV) dimension in the diastolic phase (LVDd), LV dimension in the systolic phase (LVDs), LV posterior wall thickness at end-diastole (LVPWd) and at end-systole (LVPWs) were measured with M-mode echocardiography utilizing a Vevo 2100 (Visual Sonics Inc., Ontario, Canada). LV long-axis fractional shortening (FS) and ejection fraction (EF) were calculated offline.

### Surgical preparation and synchrotron radiation (SR) microangiography

**Fig. 1:**
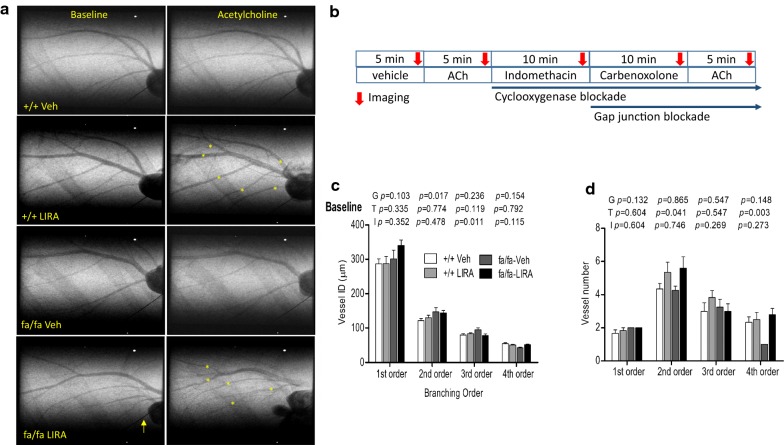
Representative synchrotron angiograms of the coronary vasculature in Zucker rats after 8 weeks on a high-salt diet. **a** Images during baseline, infusions of acetylcholine (ACh). **b** Acute infusion protocol during coronary vasculature imaging. **c** Vessel internal diameter categorized by branching order during baseline ringer’s lactate infusion. **d** Visualized vessel number during baseline. Calibration wire (yellow arrow) = 50 μm. The data are shown as the mean ± SEM; N = 5–6 rats per group. The significance of group differences was determined by ANOVA for factors of genotype (G), drug treatment (T) and their interaction (I)

Following high-salt diet treatment for 8 weeks, SR contrast microangiography was utilized to measure in vivo coronary arterial vessel internal diameters (50–350 µm) and visualized vessel number in hypertensive insulin resistant +/+ and fa/fa rats (at 16 weeks of age) [21]. Rats were induced with isoflurane (4%) and anesthesia maintained (2%) throughout surgical preparation and the imaging experiment. All rats were artificially ventilated (Model MA-01746, Harward Apparatus, Holliston, USA) and a jugular vein was cannulated with polyethylene tubing (SP-31 Natsume Seisakusho Co., Ltd, Tokyo, Japan) for blood collection and renal function analyses, fasted blood glucose (FBG) measurement (500 μL) and drug infusion during the imaging protocol (Fig. 1b). Rectal temperature was monitored and regulated at 36–37 °C using heating pad throughout the experimental procedure. The right carotid artery was cannulated for high speed injection of contrast agent and a right femoral artery cannulated for recording of arterial pressure via pressure transducer (MLT0699, AD Instruments, NSW, Australia) to determine mean arterial pressure (MAP) and heart rate (HR). Contrast medium was injected as a small arterial bolus at 15 min intervals (0.3–0.4 mL bolus, Iomeron 350, Bracco-Eisai, Tokyo, Japan). The same imaging and high-speed shutter system as described in earlier studies was used here [24]. Images (1024 × 1024 pixels) were stored in 10-bit format. In all images a 50 µm tungsten wire was included for calibration of vessel ID measurements. The input field of the SATICON X-ray detector system was 9.5 mm × 9.5 mm. Shutter open time was 0.8–1.0 ms. Monochromatic X-ray energy was adjusted to 33.2 keV, just above the iodine K-edge energy for maximal contrast. Coronary angiogram image analysis was performed as previously described [18]. At the end of the imaging rats were killed by potassium chloride solution (2 mmol/kg) infusion under deep anesthesia and the myocardium was collected for further analysis.

### SR microangiography protocol and rationale

Intraarterial injection of iodinated contrast was made during baseline conditions with a lactate infusion, then during a subsequent (1) infusion of acetylcholine (ACh, 5 µg/kg/min intravenous (i.v), (2) following combined blockade of prostaglandin production with the cyclooxygenase (COX) inhibitor indomethacin (Indo, 10 mg/kg iv bolus) and (3) uncoupling of smooth muscle gap junctions with carbenoxolone administration (carbenox, 10 mg/kg iv bolus) following a 10 min equilibration period. (4) We then assessed endothelium-dependent dilation after blockade with a repeat infusion of ACh (3.0 µg/kg/min) (Fig. 1b). Smooth muscle gap junctions are considered to be important for the distal transmission of dilation along vessels by conducted dilation, which is considered to be mediated by NO and endothelium derived hyperpolarization factors (EDHF) including small and large conductance calcium-activated potassium channels and myoendothelial gap junctions [25]. Indo + carbenox blockade was used in this study to inhibit radial dilation through prostanoids and myoendothelial gap junctions to determine if the reduced NO bioavailability due to the metabolic syndrome impaired radial and conducted dilation within coronary vasculature. Changes in internal vessel diameter (ID) or caliber of the same vessels that were visualized in all treatment periods are considered in this study to be indicative of local (radial) dilatory function. On the other hand, a change in the number of angiographically visualized vessel segments during stimulation is used here as an index of a change in distal or conducted dilation. Therefore, we expected that any significant decrease in vessel ID or lack of change in ID during stimulation of the coronary vasculature with ACh infusion would indicate that there is underlying smooth muscle/endothelial dysfunction respectively, involving impairment of NO-mediated signaling. On the other hand, a reduction in newly visualized vessels during stimulation or a lack of change in visualized vessel number would indicate that there is impairment in at least one of the mechanisms responsible for conduction of distal dilation and regulation of global perfusion (NO and EDHF).

### Assessment of vessel internal diameter and visible vessel number

Quantitative measurements of vessel internal diameter (ID) was described as previously [19, 24] using ImageJ (version 1.48, NIH, Bethesda, USA). All angiograms were under went median filtering (2-pixel radius) before vessel ID determination. Arterial vessels were categorized according to their branching order (1st, 2nd, 3rd and 4th). Percentage change in 1st to 4th order vessel caliber and visible vessel number during infusions of ACh and ACh stimulation during blockade of prostaglandin production and uncoupling of gap junctions from baseline.

### Tissue preparation and Western blot analysis

Myocardial protein samples were homogenized in ice-cold lysis buffer containing: 150 mM NaCl, 25 mM Tris–HCl (pH 7.5), 5 mM MgCl_2_ (pH 8.0) and 5% glycerol, 1% Triton X-100, complete EDTA free protease inhibitor cocktail (Roche, Indianapolis, USA). Myocardial proteins were separated by SDS–PAGE, 30–50 µg of protein was loaded on 10% sodium dodecyl sulfate–polyacrylamide gels (Bio-Rad Laboratories, CA, USA), and subsequently transferred to polyvinylidene fluoride membrane. These membranes were then blocked for 1 h with blocking one and phospho-blocking one (Nacali tesque Inc, Kyoto, Japan) at room temperature. The following primary antibodies were used to quantify the myocardial protein expression levels: anti-CD-68 (1:1000, ab-31630), anti-GLP-1R (1:1000, sc-390773), anti-nitrotyrosine (NT, 1:1000, sc-32757), anti-nuclear factor (NF) kappa B (NF-κB, 1:1000, sc-7386), atrial natriuretic peptide (ANP, 1:1000, ab-91250), osteopontin (OPN, 1:1000, sc-21742), transforming growth factor-β1 (TGF-β1, 1:1000, sc-146), endothelin-1 (ET-1, 1:1000, ab-2786), total eNOS (1:1000, sc-376751), interleukin (IL)-1β (1:1000, sc-7884) and β-actin (1:1000, sc-47778) (Santa Cruz Biotechnology (sc), CA, USA; Abcam Laboratory (ab), MA, USA). Specific antibody binding was revealed using appropriate secondary antibodies (1:10,000; #7076S; Goat anti-rabbit IgG; #7074S; Goat anti-mouse IgG, Cell Signaling Technology, Inc. MA, USA) coupled to peroxidase and with the Clarity Western ECL Substrate (#1705061; Bio-Rad Laboratories, CA, USA). Chemiluminescence signals were visualized using an Amersham Imaging System (AI-680, GE Healthcare Life Sciences, PA, USA) and were quantified using β-actin signal as a protein loading control.

### Quantitative real-time PCR

Myocardial tissue was homogenized in TRIZOL reagent (QIAGEN, CA, USA), and total RNA was extracted, and DNase were treated while purification (RNeasy Mini Purification Kit, RNase-Free DNase Set, QIAGEN, CA, USA). Reverse transcription was performed using the High-Capacity cDNA synthesis kit (#4368814, Thermo Fisher Scientific, CA, USA) as per the manufacturer’s instructions. PCR reactions were performed on a StepOne 7500 RT-PCR system using TaqMan Universal Master Mix and predesigned primers (NOX-1: Rn00586652_m1; GPx-1: Rn00577994_g1; Vcam-1: Rn00563627_m1; ET-1: Rn00561129_m1; eNOS: Rn02132634_s1; VEGF: Rn01511602_m1) obtained from Applied Biosystems (Applied Biosystems, Foster City, CA, USA). GAPDH (Rn01775763_g1) were used as normalizing internal controls. All samples were measured in duplicate. We employed the 2^−ΔΔCt^ method to analyze the genes of interest relative to GAPDH reference gene as described [26].

### Determination of glutathione concentrations

Reduced glutathione (GSH) and oxidized glutathione disulphide (GSSG) levels in plasma samples were measured using enzyme immunoassay kits (Cayman Chemical Corp., MI, USA) as per manufacturer’s instructions.

### Histology and staining

Myocardial tissue was fixed with ice-cold 4% paraformaldehyde for 48 h, and embedded in paraffin, and processed for histology as described previously [23]. Transverse sections were stained either with Azan-Mallory solution for evaluation of perivascular fibrosis or with hematoxylin–eosin solution for evaluation of cardiomyocyte hypertrophy. We determine it as the ratio of the area of fibrosis immediately surrounding the blood vessel walls to the total area of the vessel. Cross-sectional area (CSA) of cardiomyocytes were measured for 100 cardiomyocytes selected per section (400×). The mean CSA of individual rats was then pooled for group comparisons [27].

### Statistical analysis

All data are presented as mean ± SEM unless otherwise stated. Statistical analyses were performed with Graph Pad Prism software (Version 5.0, La Jolla, CA, USA). *p *< 0.05 was considered statistically significant. Normality was assessed using the Shapiro–Wilk test. The percentage change in vessel ID and the mean vessel ID of each branching order in individual rats (N = 5–6 animals per group) were pooled together for group comparisons. To compare the means of variables among the rat treatment groups we used two-way analysis of variance (ANOVA) test. Further, to evaluate significance of differences due to either genotype (G, fa/fa vs. +/+) or drug treatment (T, LIRA vs. vehicle) and their interaction (I).

## Results

### Body weights (BW) and SBP

The BW of fa/fa rats were higher than those of +/+ rats thought out the study. There were no significant BW differences in vehicle or LIRA treated groups (data not shown). SBP was modestly elevated in fa/fa rats (genotype *p *= 0.018) in comparison to the +/+ rats, however, LIRA did not significantly salter SBP (Table 1).

**Table 1:** Animal characteristics, heart mass and renal function of Zucker rats after LIRA treatment on a high-salt diet

Parameter	Group				2-way ANOVA *p* value		
	+/+ Veh	+/+ LIRA	fa/fa Veh	fa/fa LIRA	Genotype	Treatment	Interaction
BW (g)	322.1 ± 5.3	324.3 ± 5.8	518.0 ± 7.6	530.4 ± 14.8	< 0.0001	0.475	0.581
BG (mg/dL)	11.7 ± 0.3	11.1 ± 0.3	13.3 ± 0.9	10.7 ± 0.6	0.211	0.023	0.148
SBP (mmHg)	125.9 ± 8.5	122.0 ± 2.9	132.6 ± 7.3	123.5 ± 3.2	0.018	0.366	0.182
HW (mg)	764.5 ± 22.9	755.1 ± 12.0	922.4 ± 49.2	898.2 ± 17.3	0.0001	0.473	0.945
HW/BW ratio	2.37 ± 0.05	2.33 ± 0.07	1.78 ± 0.09	1.69 ± 0.01	< 0.0001	0.317	0.897
GFR (mL/min)	1.57 ± 0.08	1.52 ± 0.05	0.98 ± 0.19	1.28 ± 0.05	0.018	< 0.0001	< 0.0001
Albumin (mg/L)	10.0 ± 1.2	10.1 ± 1.2	75.4 ± 11.6	67.1 ± 14.1	< 0.0001	0.665	0.653
Creatinine (mg/L)	60.0 ± 6.5	76.8 ± 4.0	25.3 ± 4.1	22.2 ± 3.2	< 0.0001	0.106	0.069
A/C ratio (mg/g)	17.7 ± 3.2	13.3 ± 1.8	327.0 ± 71.1	285.9 ± 46.1	< 0.0001	0.433	0.499
BUN (mg/dL)	13.9 ± 1.1	13.9 ± 0.6	16.7 ± 0.6	14.2 ± 0.5	0.061	0.032	0.110
Serum albumin (g/dL)	2.85 ± 0.1	2.86 ± 0.1	3.62 ± 0.2	3.64 ± 0.05	< 0.0001	0.189	0.033

*A/C* albumin creatinine ratio, *BG* blood glucose, *BUN* blood urea nitrogen, *BW* body weight, *GFR* glomerular filtration rate, *HW* heart weight, *HW/BW* ratio of heart weight to body weight, *SBP* systolic blood pressure. Age-matched +/+ Veh, lean rats treated with vehicle; +/+ LIRA, lean rats treated with LIRA (0.1 mg/kg/day); fa/fa Veh, obese rats treated with vehicle; fa/fa LIRA, obese rats treated with LIRA (0.1 mg/kg/day)

### LIRA effect on renal function and glycemic profile

Urinary albumin, albumin-creatinine ratio, and serum albumin values were significantly different between the fa/fa and +/+ rats (genotype *p *< 0.0001, Table 1). These indices of renal dysfunction were significantly upregulated in vehicle-treated fa/fa rats when compared to +/+ rats. LIRA did not consistently alter the elevated albumin in fa/fa rats, but there was a trend for creatinine to be reduced by LIRA in fa/fa (interaction *p *= 0.069) and BUN was lowered by LIRA (Table 1). GFR was impaired in vehicle-treated fa/fa rats (genotype *p *= 0.018) relative to +/+ rats, but was nearly normalized in LIRA-treated fa/fa rats (drug and interaction *p *< 0.0001). In contrast, LIRA treatment did not improve creatinine clearance in fa/fa LIRA rats compared to those of vehicle-treated fa/fa rats (*p *= 0.433). Vehicle-treated fa/fa rats developed mild hyperglycemia as their BG levels were a little elevated compared to those of +/+ rats (genotype *p *= 0.211) which showed prediabetic BG levels presumably due to the high-salt diet exposure and possibly isoflurane conditions during blood collection. LIRA treatment attenuated the increase in BG level in fa/fa rats (treatment *p *= 0.023; Table 1).

### LIRA treatment improved myocardial function

As shown in Table 2, interventricular septum thickness at end-diastole (IVSd) (*p *= 0.003) was significantly greater than those of +/+ rats. However, LIRA treatment did not reduce interventricular thickness (treatment *p *= 0.903) relative to vehicle-treated fa/fa rats. However, there was no such genotype trend observed in interventricular septum thickness at end-systole (IVSs) (genotype *p *= 0.102; treatment *p *= 0.255). Left ventricle (LV) posterior wall in diastole (LVPWd) (genotype *p *= 0.059) but not LV posterior wall in systole (LVPWs) (genotype *p *= 0.556) of fa/fa were larger compared to those of +/+ rats. LV internal diameter at end diastole (LVIDd) (genotype *p *= 0.033) and LV internal diameter at end systole (LVIDs) (genotype *p *= 0.007) in fa/fa rats were larger compared with +/+ rats. However, LIRA treatment did not reduce these dimensions significantly, when compared to those of vehicle-treated rats [treatment *p *= 0.953 (LVIDd); *p *= 0.960 (LVIDs)]. However, EF, FS and cardiac output were did not improved in LIRA treatment [treatment *p *= 0.688 (EF); *p *= 0.658 (FS) and *p *= 0.123 (CO)] (Table 2).

**Table 2:** Echocardiographic parameters of Zucker rats after LIRA treatment on a high-salt diet

Parameter	Group				2-way ANOVA *p* value		
	+/+ Veh	+/+ LIRA	fa/fa Veh	fa/fa LIRA	Genotype	Treatment	Interaction
HR (bpm)	435 ± 28	452 ± 42	369 ± 22	375 ± 33	< 0.0001	0.191	0.416
IVSd (mm)	1.81 ± 0.14	1.87 ± 0.08	2.35 ± 0.10	2.42 ± 0.21	0.003	0.903	0.719
IVSs (mm)	2.91 ± 0.11	3.09 ± 0.25	3.27 ± 0.13	3.08 ± 0.11	0.102	0.255	0.828
LVIDd (mm)	6.17 ± 0.10	6.21 ± 0.23	6.76 ± 0.18	6.73 ± 0.26	0.033	0.953	0.941
LVIDs (mm)	2.46 ± 0.26	2.55 ± 0.43	3.87 ± 0.41	3.42 ± 0.19	0.007	0.960	0.249
LVPWd (mm)	2.37 ± 0.21	2.27 ± 0.25	2.63 ± 0.31	2.72 ± 0.20	0.059	0.800	0.912
LVPWs (mm)	3.42 ± 0.15	3.58 ± 0.25	3.30 ± 0.15	3.33 ± 0.17	0.556	0.589	0.705
EF (%)	90.63 ± 0.99	89.70 ± 1.39	82.96 ± 1.49*	84.09 ± 2.15*	0.0007	0.688	0.272
FS (%)	63.53 ± 1.56	62.69 ± 2.22	53.49 ± 1.90*	55.17 ± 2.50*	0.0006	0.658	0.211
CO (mL/min)	9.46 ± 0.41	10.53 ± 0.71	7.91 ± 0.93	9.22 ± 0.71	0.028	0.123	0.888

*HR* heart rate, *IVSd* interventricular septal thickness at end-diastole, *IVSs* interventricular septal thickness at end-systole, *LVIDd* left ventricular (LV) internal dimension at end-diastole, *LVIDs* LV internal dimension at end-systole, *LVPWd* LV posterior wall thickness at end-diastole, *LVPWs* LV posterior wall thickness at end-systole, *EF* ejection fraction, *FS* fractional shortening, *CO* cardiac output. Age-matched +/+ Veh, lean rats treated with vehicle; +/+ LIRA, lean rats treated with LIRA (0.1 mg/kg/day); fa/fa Veh, obese rats treated with vehicle; fa/fa LIRA, obese rats treated with LIRA (0.1 mg/kg/day)

*p < 0.05 vs lean rats

### Baseline vessel internal diameter and visible vessel number

Representative synchrotron angiograms of the coronary vasculature of Zucker +/+ and fa/fa rats are shown during baseline and ACh infusions (Fig. 1a). Vessel ID in vehicle-treated +/+ rats were comparable with LIRA treated +/+ rats across 1st order to 4th order branches of the arterial vessels. In contrast, fa/fa rats had larger diameter 2nd order than +/+ (genotype *p *= 0.017) while 3rd order in LIRA treated fa/fa rats (interaction *p *= 0.011) (Fig. 1c). A similar number of 1st order arterial vessel segments were visualized in all groups, while more 2nd and 4th order vessels were observed in LIRA-treated rats (Fig. 1d) (treatment *p *= 0.041 and *p *= 0.003). MAP during baseline under anesthesia was not different between rat groups while mean heart rate (MHR) was slightly lower in fa/fa rats (Fig. 3a).

### LIRA treatment improved vessel responses to endothelium-dependent ACh stimulation

**Fig. 2:**
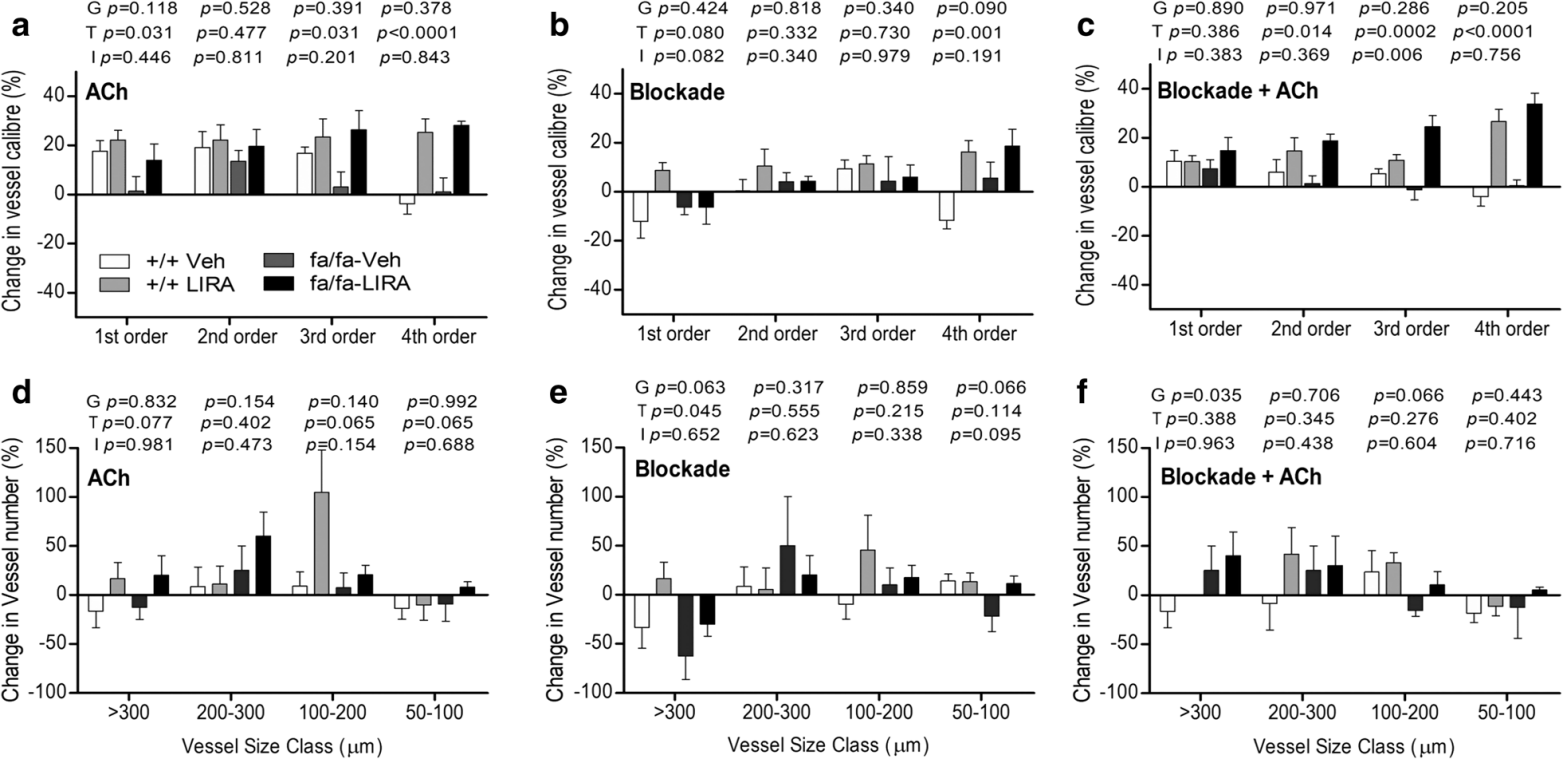
LIRA treatment improved the capacity to increase perfused segments by NO-mediated dilation in Zucker fa/fa and +/+ rats after 8 weeks on a high-salt diet. **a**–**d** Percentage change in 1st to 4th order vessel caliber and visible vessel number during infusions of ACh and ACh stimulation during blockade of prostaglandin production and uncoupling of gap junctions from baseline. Mean ± SEM. The significance of group differences was determined by ANOVA for factors of genotype (G), drug treatment (T) and their interaction (I)

**Fig. 3:**
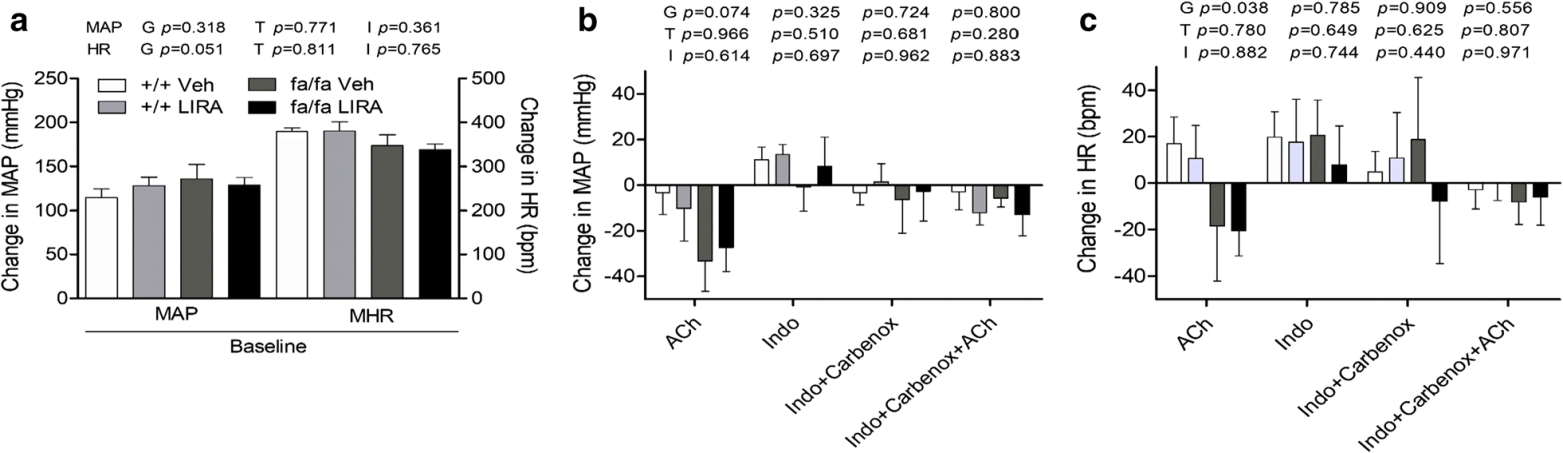
Mean arterial pressure (MAP) and mean heart rate (MHR) and their change during the acute infusion. **a** Change in MAP and change in MHR during baseline and **b**, **c** infusions of ACh, indomethacin + carbenoxolone (blockade) and post-blockade + ACh relative to vehicle Ringer’s lactate solution (baseline). Mean ± SEM. N = 5–6 rats per group. The significance of group differences was determined by 2-way ANOVA for factors of genotype (G) and drug treatment (T) and their interaction (I)

ACh stimulation evoked a greater increase in vessel ID of LIRA treated rats across all branching orders but the 2nd order (treatment 1st—*p *= 0.031; 2nd—*p *= 0.477; 3rd—*p *= 0.031; 4th—*p *= 0.0001), that was significantly greater than vehicle-treated rats, which generally did not show dilation in the 4th order branching vessels (Fig. 2a). However, the changes in visualized vessel number from large arteries to arterioles were minor and inconsistent, with a tendency to be slightly more visualized in LIRA treated rats (Fig. 2a, d). Thus, LIRA treatment primarily restored the dilatory response to ACh in the microvessels of fa/fa rats. There was a trend toward a larger decrease in MAP in fa/fa rats in response to ACh compare with +/+ rats (genotype *p *= 0.074; Fig. 3b). However, LIRA treated rats did not differ significantly in the size of the MAP change from vehicle-treated rats. Hence the increased dilator ACh response in LIRA treated rats was not due to a difference in arterial pressure change.

### LIRA treatment improved vessel response during prostaglandin and gap junction blockade and subsequent ACh stimulation

Blockade of COX and gap junctions had little influence on the calibre of large to medium sized vessels in the 1st to 3rd order branches, but was increased significantly in 4th order branches of LIRA treated rats (treatment *p *= 0.001; Fig. 2b). The reduction in visualized number of large arteries (> 300 µm class) during blockade was partially alleviated by LIRA treatment mainly in the +/+ rats (treatment *p *= 0.045) (Fig. 2e). Notably, ACh-mediated vasodilation post blockade was of a similar magnitude to ACh stimulation prior to blockade in 2nd to 4th order branches in LIRA treated rats (Fig. 2c). Paradoxically, there was a small increase in the number of visualized large arteries > 300 µm in both fa/fa rat groups, a trend for a reduction in small arteries (100–200 μm class), otherwise there was little change in the number of visualized microvessels across all groups (Fig. 2f). Hence, transmission of endothelium-dependent radial dilation (calibre increase) to more distal resistance coronary vascular segments that were consistently visible from the baseline was little affected by blockade of smooth muscle gap junctions in the LIRA treated rats but impaired in vehicle treated lean and obese rats.

### LIRA treatment suppressed the early stages of myocardial inflammation

**Fig. 4:**
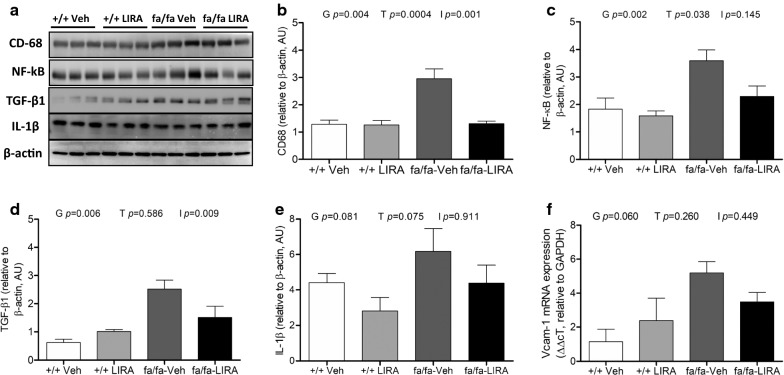
The effects of LIRA treatment on expression of CD-68, NF-κB, TGF-β1 and IL-1β in the left ventricle. **a** Protein expression levels of CD-68, NF-κB, TGF-β1 and IL-1β detected by Western blotting. β-actin was used as an internal control. Bands are representative of four separate experiments. **b**–**e** Relative density values of CD-68, NF-κB, TGF-β1 and IL-1β. **f** mRNA expression of Vcam-1 in Zucker rat myocardium. GAPDH gene was used as an internal control. The ΔΔcT values are shown as the mean ± SEM. N = 5–6 rats per group. The significance of group differences was determined by ANOVA for factors of genotype (G), drug treatment (T) and their interaction term (I)

Compared with +/+ rats, protein expressions of CD-68, NF-κB, IL-1β and TGF-β1 were upregulated in vehicle-treated fa/fa rats (genotype *p *< 0.05; Fig. 4). LIRA treatment significantly decreased expression of CD-68 (treatment *p *< 0.001), NF-κB (treatment *p *= 0.038) tended to decrease and IL-1β (treatment *p *= 0.075) and a significant genotype-drug interaction (*p *= 0.009) indicated TGF-β1 protein expression was only attenuated in the fa/fa rats by LIRA treatment (Fig. 4a–d). In addition, mRNA levels of Vcam-1 showed a strong trend to be increased (genotype *p *= 0.06) in fa/fa rats (Fig. 4f). Hence, LIRA treatment reduced expression of the master regulator NF-κB and induction of downstream inflammatory pathways.

### LIRA treatment reduced myocardial oxidative stress but not systemic oxidized glutathione levels

**Fig. 5:**
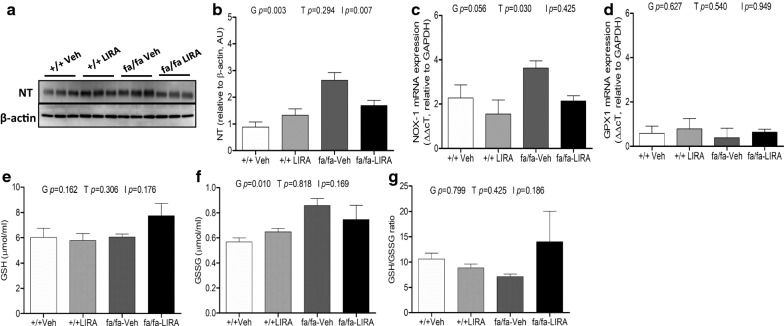
The effects of LIRA treatment on nitrosative, oxidative stress marker and anti-oxidant levels. **a** Protein expression levels of NT in left ventricle detected by Western blotting. β-actin was used as an internal control. Bands are representative of two separate experiments. **b** Relative density values of NT. **c**, **d** mRNA expressions of NOX-1 and Gpx-1 in Zucker rat myocardium. GAPDH gene was used as an internal control. The ΔΔcT values are shown as the mean ± SEM. Effects of LIRA on Reduced GSH (**e**), oxidized GSSG (**f**), and GSH/GSSG ratio (**g**). N = 5–6 rats per group. The significance of group differences was determined by ANOVA for factors of genotype (G), drug treatment (T) and their interaction term (I)

Compared with +/+ rats, NT protein expression was upregulated in fa/fa rats (genotype *p *= 0.003), but more attenuated by LIRA treatment (interaction *p *= 0.007) in the fa/fa rats (Fig. 5a, b). In addition, there was a strong trend for mRNA levels of Nox-1 to be increased (genotype *p *= 0.056) in fa/fa rats, and reduced by LIRA (treatment *p *= 0.030) (Fig. 5c), Gpx-1 mRNA expression showed no significant differences between rat groups (Fig. 5d).

Reduced GSH plasma concentration was not different between the vehicle treated +/+ and fa/fa rats (Fig. 5e). Importantly, the concentration of oxidized GSSG levels was elevated in fa/fa rats in comparison to +/+ rats (genotype *p *= 0.010), but LIRA treatment did not consistently reduce this level (treatment *p *= 0.818; Fig. 5f). Consequently, there was no difference in GSH/GSSG ratio (Fig. 5g).

### LIRA treatment effects on ET-1/NO signaling pathways and GLP-1R expression

**Fig. 6:**
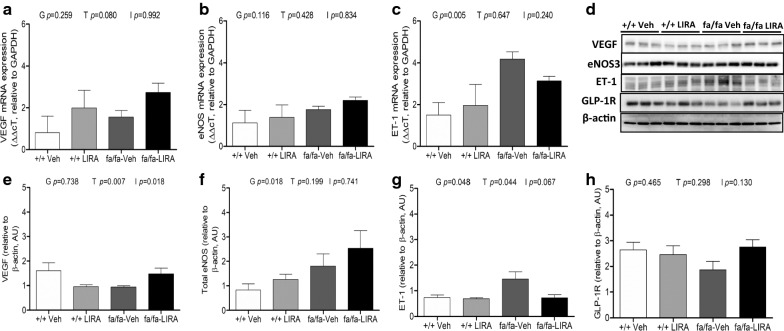
Changes in mRNA abundance of genes related to NO-signaling pathway. **a**–**c** mRNA expressions of VEGF, eNOS and ET-1 in Zucker rat myocardium. GAPDH gene was used as an internal control. The ΔΔcT values are shown as the mean ± SEM. **d**–**h** The effects of LIRA treatment on expression of VEGF, total-eNOS, ET-1 and GLP-1R in the left ventricle. **d** Protein expression levels of VEGF, total-eNOS, ET-1 and GLP-1R detected by Western blotting. β-actin was used as an internal control. Bands are representative of five separate experiments. **e**–**h** Relative density values of VEGF, total-eNOS, ET-1 and GLP-1R. N = 5–6 rats per group. The significance of group differences was determined by ANOVA for factors of genotype (G), drug treatment (T) and their interaction term (I)

The profibrotic and vasoconstrictor factor ET-1 was elevated at both mRNA (genotype *p *= 0.005) and protein (genotype *p *= 0.048) levels in fa/fa rats, while eNOS and VEGF mRNA expression levels did not differ between +/+ and fa/fa rats (Fig. 6a–c, g). At the protein level total eNOS expression was greater in fa/fa rats than +/+ rats (genotype *p *= 0.018), but this was not significantly altered by LIRA (Fig. 6d, f). While VEGF mRNA tended to be increased by LIRA (treatment *p *= 0.08), VEGF protein expression was unexpectedly suppressed in the +/+ and increased in fa/fa relative to vehicle counterparts (treatment *p *= 0.007; interaction *p *= 0.018; Fig. 6a, d–e). Although myocardial GLP-1R protein expression appears to be mildly depressed in vehicle-treated fa/fa rats there was no significant difference between genotypes (*p *= 0.465; Fig. 6d, h).

### LIRA treatment partially attenuated perivascular fibrosis and myocardial remodeling markers

**Fig. 7:**
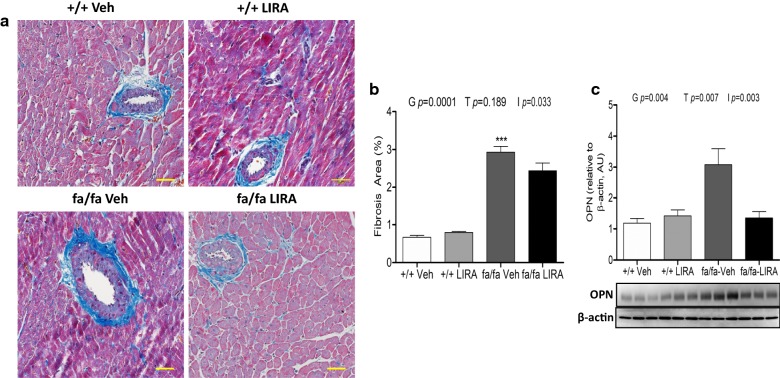
Azan-Mallory staining for perivascular collagen expression. **a** Azan-Mallory stained myocardial sections from a vehicle-treated +/+ and LIRA-treated +/+ rats showing low levels of collagen deposition (blue fibrils); broad adventitial ring of strong blue fibrotic tissue reactivity in a vehicle-treated fa/fa rats; LIRA-treated fa/fa rats showing moderate blue fibrous tissue positivity around a coronary artery. Scale bar are 20 µm. **b** Quantification of perivascular fibrosis (%). **c** Protein expression levels of OPN detected by Western blotting. β-actin was used as an internal control. Bands are representative of two separate experiments. Relative density values of OPN, data are shown as the mean ± SEM, N = 5–6 rats per group. The significance of group differences was determined by ANOVA for factors of genotype (G), drug treatment (T) and their interaction term (I)

Azan-Mallory staining revealed that fibrosis in perivascular regions of the myocardium was increased significantly in fa/fa rats compared with +/+ rats (genotype *p *= 0.0001), but this increase in perivascular fibrosis was only partially attenuated in LIRA treated fa/fa rats compared with vehicle-treated fa/fa rats as the drug treatment was not significant, but the interaction term was (p = 0.033) (Fig. 7a, b). Nevertheless, protein levels of OPN in the LV were significantly greater in fa/fa rats than in +/+ rats (genotype *p *= 0.004) and this expression was normalized by LIRA treatment (treatment *p *= 0.007; interaction *p *= 0.003; Fig. 7c).

### LIRA treatment improved myocardial hypertrophy

**Fig. 8:**
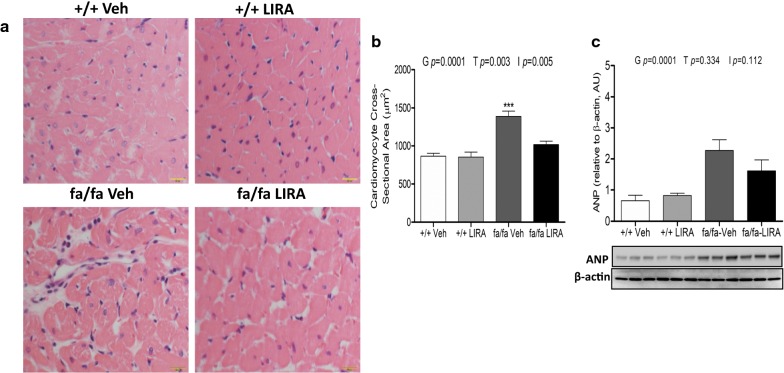
**a** Hematoxylin and eosin stained sections of myocardium illustrating cardiomyocytes in cross-section (×400). Scale bars are 20 µm. **b** Bar graph showing quantitative analysis of cross -sectional area of cardiomyocytes. **c** The effects of LIRA treatment on expression of ANP in left ventricle. **c** Protein expression levels of ANP detected by Western blotting. β-actin was used as an internal control. Bands are representative of two separate experiments. Relative density values of ANP, data are shown as the mean ± SEM, N = 5–6 rats per group. The significance of group differences was determined by ANOVA for factors of genotype (G), drug treatment (T) and their interaction term (I)

Compared with +/+ rats, fa/fa rats showed marked myocardial hypertrophy including increased HW and cardiomyocyte cross sectional area (genotype *p *= 0.0001) (Table 1; Fig. 8a, b). LIRA treated fa/fa rats exhibited less myocardial hypertrophy characterized by decreased cardiomyocyte cross sectional area (treatment *p *= 0.003, interaction *p *= 0.005), when compared with those of vehicle-treated fa/fa rats and +/+ rats, but this did not alter heart mass or HW/BW relative to the vehicle-treated fa/fa rats (Table 1; Fig. 8a, b). Paralleling the difference in heart mass and cardiomyocyte cross sectional area, myocardial protein levels of ANP in fa/fa rats were elevated compared with the +/+ rats (genotype *p *= 0.0001). However, LIRA treatment did not attenuate ANP expression significantly (Fig. 8c).

## Discussion

Previous studies showed that a high Na^+^ diet was closely associated with hypertension, insulin resistance, systemic inflammation and oxidative stress, which thereby increased the incidence of cardiovascular events [28, 29]. In the present study, we investigated whether LIRA treatment improved endothelial vasodilator function across the coronary macro and microcirculations and if it prevented cardiac remodeling in a rat model of metabolic syndrome exacerbated by chronic exposure to a high Na^+^ diet. Herein, we showed that LIRA treatment improved NO-mediated dilation in the coronary small arterial and arteriolar vessels, while reducing myocardial ET-1 protein expression in Zucker obese rats. Furthermore, LIRA treatment normalized cardiomyocyte cross sectional area but only partially reduced the development of perivascular fibrosis in association with reduced expression of protein markers of remodeling, including OPN and TGF-β1. Taken together, these results suggest that LIRA treatment greatly attenuated the progression of coronary dysfunction, partially though decreased nitrosative stress and ET-1 production and in part by improving endothelium dependent NO-mediated dilation in small arteries and arterioles.

The integrity and function of the endothelium is critical for the tonic regulation of blood flow, primarily through the release of vasoconstrictor and vasodilator factors that alter the calibre of vessels through their actions on underlying smooth muscle that determines local vascular tone in each segment. Endothelial dysfunction is an early sign of coronary vascular disease and impaired endothelium-dependent relaxation to vasodilator agonists such as ACh is a characteristic clinical feature of endothelial dysfunction that has also been observed in both conduit and resistance arteries of experimental models of diabetes and hypertension [30–32]. Endothelial dysfunction is often triggered by a reduced protein expression of eNOS and impaired eNOS phosphorylation at the serine 1177 residue, decreased availability of eNOS substrate and or co-factors, all of which contribute to decrease NO bioavailability. In the present study, we showed that LIRA improved ACh-mediated vasodilation I small artery and arterioles (< 150 μm diameter) in rats with metabolic syndrome that was exacerbated by chronic exposure to a high-salt diet (Figs. 1a and 2a, d). Since the ACh-mediated dilatory response was of similar magnitude in the LIRA rats before and after blockade of dilatory prostaglandins we reason that this response primarily involves NO release and to some extend EDHF. Importantly, our assays of myocardial protein expression also support the suggestion that LIRA prevented the decline of NO and EDHF in the microvessels through increased eNOS and VEGF protein and decreased ET-1 protein (Fig. 6). These results suggest that LIRA treatment improved the capacity for dilation of micro vessels and hence, improved myocardial perfusion by restoring the balance of tonic ET-1/NO production. Similarly, we showed utilizing the same rat model that improvement in endothelium mediated NO-dependent dilation extends to small arteries and arterioles of the renal circulation [21]. In support of these studies, it has also been established that LIRA increases NO-bioavailability in the vasculature in other disease states including arterial stenosis [33]. Further, Aung et al. [34] recently showed that local delivery of LIRA increased skin microvascular perfusion in diabetic subjects and increased supernatant nitrate concentration and eNOS phosphorylation in human microvascular endothelial cell (HCMEC/D3 cell line) culture. Taken together, these findings suggest that it is reasonable to conclude that the improvement in coronary microvascular perfusion that we observed in LIRA treated obese Zucker rats is in part due to increased coronary NO production.

Multiple experimental studies have demonstrated that GLP-1 agonist treatment has anti-inflammatory effects on endothelial cells and monocytes through inhibition of inflammatory cascades involving vascular cell adhesion molecules and intracellular adhesion molecules [35–37]. In addition, a recent clinical study suggest that LIRA treatment decreases endoplasmic reticulum stress in endothelial cells and restores insulin-mediated endothelial NO production in patients with T2DM [38]. In experimental diabetic animal model, LIRA treatment suppressed PCSK9 expression in HepG2 cells through HNF1α-dependent mechanism and decreased LDLR [39]. Further, db/db mice fed with high fat diet improved hepatic lipid accumulation by promoting reversal of cholesterol transport [40]. In our model of metabolic syndrome, Vcam-1 mRNA expression showed a strong trend to be reduced by LIRA in the kidney [21] but not in the myocardium [13]. On the other hand, markers of macrophage infiltration (CD68 protein expression and CD+ cell density in sections) suggest that LIRA greatly suppressed macrophage infiltration in both the heart and kidney in our studies. Furthermore, there was a strong trend for the somewhat elevated levels of the early potent inducer of the inflammatory cascade IL-1β to be suppressed by LIRA in obese rats. In line with this study, others have shown that LIRA treatment improves endothelial function and cardiac function through suppression of oxidative stress and inflammation [41, 42].

Oxidative stress refers to the imbalance between overwhelming oxidative processes and the depressed activity of anti-oxidant pathways resulting in excess accumulation of reactive oxygen species (ROS) and reactive nitrogen species in vivo, causing the oxidation of proteins [5, 43]. Oxidative stress damages the endothelium and impairs production of vasodilators such as NO as a result of oxidation of co-factor tetrahydrobiopterin, while increasing vasoconstrictor factors. Importantly, others have already shown that LIRA and other GLP-1 agonists reduce oxidative stress in diabetic rats and T2DM patients [17, 20, 44, 45]. However, in this study we found only small improvements in our assessment of oxidative stress and nitrosative stress imbalance. Notably, we observed a significant reduction in NT, while Nox1 gene transcription was also reduced (Figs. 4a, b and 5a–c). These findings suggest that nitrosative stress at least was reduced along with monocyte/macrophage infiltration of the myocardium, which would be expected to reduce reactive nitrogen species generation in the matrix. However, LIRA did little in this setting of metabolic syndrome combined with a high Na^+^ diet to reduce the overall redox imbalance. There was no significant improvement at this LIRA dose in the reduced GSH form of and no attenuation of GSSG accumulation (Fig. 5e–g). Further, Gpx-1 transcription was not significantly altered. The lack of an effect of LIRA on ROS generation might therefore be related to the low dose regime.

Previous studies have suggested that TGF-β plays a major role in the development of hypertension [8, 46]. High dietary salt intake is a potent stimulus for the vascular production of TGF-β, which increased the NOX-4 in the endothelium and thereby decreased the NO bioavailability due to the increased ROS in the vascular wall leading to increased vascular tone [7]. In the current study, no significant effect of LIRA on SBP was detected. However, in our hands, LIRA significantly lowered SBP in our previous study with the same rat model [21]. In both studies the LIRA normalized the impairment of renal function to a similar extent. Oxidative stress is a driving factor in myocardial hypertrophy evoked by exposure to a high-salt diet [47, 48]. Another group has reported that LIRA administration diminished myocardial fibrosis and myocardial hypertrophy through the downregulation of TGF-β expression in obese diabetic rats [46, 49]. However, the current study clearly demonstrates that while this dose of LIRA prevents cardiomyocyte enlargement by inhibiting ET-1, OPN and TGF-β signaling (Figs. 7 and 8), it was not sufficient to normalize heart mass at the end of the treatment period in these young adult male rats with metabolic syndrome. We cannot determine from these findings whether the latter was due to ongoing excess oxidative stress, the hearts were already hypertrophied at the start of treatment (8 weeks of age), or the combined influence of these factors. Possibly for the same reasoning, perivascular fibrosis was only partially reduced by our LIRA dose regime in these young adult rats.

## Conclusion

**Fig. 9:**
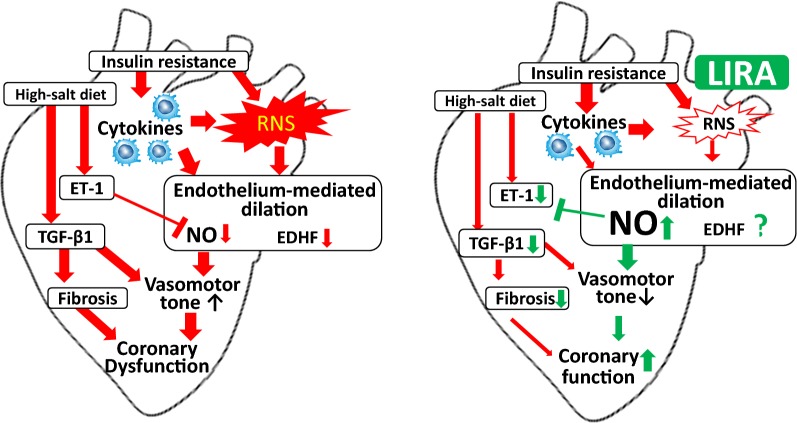
Proposed schematic representation for the beneficial effects of LIRA in coronary dysfunction associated with the additive effects of obesity and a high-salt diet. High-salt diet induced coronary dysfunction is characterized by increased RNS-production, inflammation and reduced NO bioavailability. LIRA treatment improved endothelial function, oxidative stress and inflammation. These effects are mediated through the downregulation of ET-1, TGF-β1 and upregulation of eNOS and to a variable extent VEGF

Our results demonstrate that high-salt diet feeding combined with metabolic syndrome evoked impairment of the coronary microvessel vasodilator function and sustained GLP-1R activation with LIRA improved the capacity for NO-mediated dilation to normalize the regulation of myocardial perfusion (Fig. 9). Importantly, LIRA treatment restored endothelial function of coronary arterioles through increasing NO-bioavailability as well as reducing the induction of inflammation in Zucker obese rats. It remains to be established if LIRA is equally protective in established diabetes combined with hypertension.

### Study limitations

A limitation of the present work is that experiments were performed with a low dose of LIRA, however, based on recent published studies [16, 19] further studies are warranted at higher doses. We predict that a higher dose might more completely inhibit inflammation and oxidative stress than in the present study. Further, the current study was designed to investigate if NO bioavailability is altered by LIRA, however, we did not have the opportunity to evaluate the potential contributions of EDHF to the improved regulation of coronary perfusion.

## Data Availability

The datasets used and/or analyzed in the current study are available from the corresponding author upon reasonable request.

## Abbreviations

A/C: Albumin creatinine ratio
ACh: Acetylcholine
ANP: Atrial natriuretic peptide
BUN: Blood urea nitrogen
BW: Body weight
COX: Cyclo-oxygenase
EDHF: Endothelium derived hyperpolarising factors
eNOS: Endothelial nitric oxide synthase
ET1: Endothelin-1
FBG: Fasted blood glucose
FITC: Fluorescein isothiocyanate
GFR: Glomerular filtration rate
GLP-1: Glucagon like peptide-1
HR: Heart rate
HW: Heart weight
HW/BW: Ratio of heart weight to body weight
ID: Internal diameter
IVSd: Interventricular septal thickness at end-diastole
IVSs: Interventricular septal thickness at end-systole
LIRA: Liraglutide
LV: Left ventricle
LVIDd: LV internal dimension at end-diastole
LVIDs: LV internal dimension at end-systole
LVPWd: LV posterior wall thickness at end-diastole
LVPWs: LV posterior wall thickness at end-systole
MAP: Mean arterial pressure
NF-κB: Nuclear factor-kappa B
NIC: Non-invasive clearance device
NO: Nitric oxide
NOS: Nitric oxide synthase
NT: Nitrotyrosin
OPN: Osteopontin
SBP: systolic blood pressure
SEM: Standard error of the mean
SR: Synchrotron radiation
T2DM: Type-2 diabetes mellitus
TGF-β1: Transforming growth factor beta
